# Endothelin ET_B_ Receptor-Mediated Astrocytic Activation: Pathological Roles in Brain Disorders

**DOI:** 10.3390/ijms22094333

**Published:** 2021-04-21

**Authors:** Yutaka Koyama

**Affiliations:** Laboratory of Pharmacology, Kobe Pharmaceutical University, 4-19-1 Motoyama-Kita Higashinada, Kobe 668-8558, Japan; koyama-y@kobepharma-u.ac.jp

**Keywords:** endothelin, reactive astrocyte, ET_B_ receptor, Alzheimer’s disease, brain ischemia, neuropathic pain, traumatic brain injury

## Abstract

In brain disorders, reactive astrocytes, which are characterized by hypertrophy of the cell body and proliferative properties, are commonly observed. As reactive astrocytes are involved in the pathogenesis of several brain disorders, the control of astrocytic function has been proposed as a therapeutic strategy, and target molecules to effectively control astrocytic functions have been investigated. The production of brain endothelin-1 (ET-1), which increases in brain disorders, is involved in the pathophysiological response of the nervous system. Endothelin B (ET_B_) receptors are highly expressed in reactive astrocytes and are upregulated by brain injury. Activation of astrocyte ET_B_ receptors promotes the induction of reactive astrocytes. In addition, the production of various astrocyte-derived factors, including neurotrophic factors and vascular permeability regulators, is regulated by ET_B_ receptors. In animal models of Alzheimer’s disease, brain ischemia, neuropathic pain, and traumatic brain injury, ET_B_-receptor-mediated regulation of astrocytic activation has been reported to improve brain disorders. Therefore, the astrocytic ET_B_ receptor is expected to be a promising drug target to improve several brain disorders. This article reviews the roles of ET_B_ receptors in astrocytic activation and discusses its possible applications in the treatment of brain disorders.

## 1. Introduction

Astrocytes are the most abundant glial cells in the brain and play roles in supplying nutrients to nerve cells, in reinforcing synaptic structures at nerve endings, and in supporting the limited entry of blood components [1,2]. Under physiological conditions, astrocytes modulate neurotransmission through the clearance of neurotransmitters from the synaptic cleft; maintenance of extracellular fluid ion concentration; and release of gliotransmitters, such as ATP, D-serine, and L-glutamate. In brain disorders, astrocytes alter their phenotype to a reactive phenotype. Reactive astrocytes, which are characterized by hypertrophy of cell bodies with increased expression of glial fibrillary acidic protein (GFAP), are highly proliferative and often form glial scars at the damaged areas of nerve tissues. Reactive astrocytes produce various bioactive factors that regulate the pathophysiological responses of the injured nervous system [3,4]. Cytokines and chemokines produced by reactive astrocytes promote neuroinflammation and exacerbate nervous system damage [5,6]. Conversely, increased production of neurotrophic factors supports survival and synaptic formation of damaged neurons as well as neurogenesis from neural progenitor cells [7,8,9,10], which underlie the recovery of impaired nerve function. Thus, reactive astrocytes have both detrimental and beneficial effects on the injured nervous system. Therefore, it has been proposed that the regulation of astrocytic functions can lead to improvements in several brain disorders [11,12,13,14,15]. Given this background, the factors that regulate astrocytic activation have been investigated. Endothelin (ET) is a family of peptides that are also potent vasoconstrictors [16,17]. ETs are also present in nervous tissue. ET-1 production in the brain is increased during many neurological conditions such as ischemic stroke [18,19,20,21,22,23], traumatic brain injury (TBI) [24,25], Alzheimer’s disease (AD) [26], amyotrophic lateral sclerosis [27], multiple sclerosis [28], and viral infection [29,30]. Increased ET-1 in the brain causes various pathophysiological reactions in the injured nervous system, including exacerbation of neuroinflammation [31], vasospasm-mediated ischemic injury [32], angiogenesis [33], and neurogenesis [34,35,36]. Endothelin B (ET_B_) receptors are highly expressed in astrocytes [37,38]. Administration of a selective ET_B_ agonist into rat brains promoted the induction of reactive astrocytes [39], whereas ET_B_ antagonists reduced it in animal models of brain injury [40,41,42]. Therefore, the ET_B_ receptor is expected to be a target molecule to control the functions of astrocytic activation. In addition, examinations to improve brain disorders by modulating ET_B_-receptor-mediated signal have being conducted. As such, this article reviews the roles of ET-1/ET_B_ receptor signals in astrocytic activation and discusses the possibility of ET_B_ receptor agonists and antagonists being beneficial for some brain disorders.

## 2. Overview of Endothelin

### 2.1. Endothelin Ligands

The ET peptide family is composed of three isoforms of 21 amino acid cyclic peptides that are the product of different genes. Since its discovery in 1989 as a novel family of peptides produced by the vascular endothelium [16], the function of ET has attracted attention due to its role in the circulatory system because of its strong vasoconstrictor effect. However, shortly after discovery, ET ligands were shown to be present in various tissues, including the central nervous system, and have a wide range of functions, such as cell proliferation, differentiation, and neurotransmission [17]. ET-1 was the subtype discovered first and is expressed in many tissue types, including nerve tissues. Some studies have shown that the expression of ET-2 is largely limited to the gastrointestinal tract, sex organs, and pituitary gland [43,44,45]. Chang et al. found that ET-2 is involved in energy homeostasis, thermoregulation, and the maintenance of lung morphology and function [46]. ET-3 is abundantly expressed in the intestine, pituitary gland, and brain [47]. Genetic deficiency of ET-3 function in the gastrointestinal tract is associated with the development of Hirschsprung disease [48,49].

**Figure 1:**
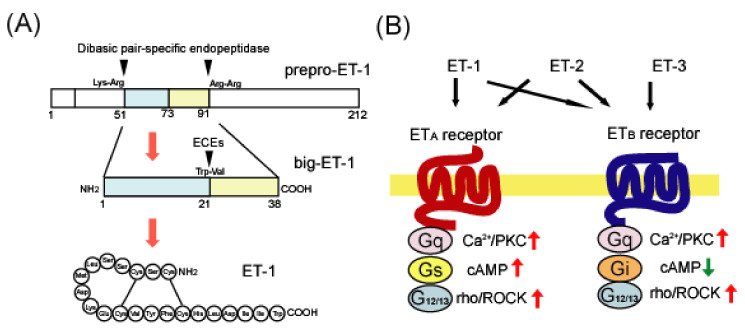
(**A**) Biosynthesis of human endothelin-1 (ET-1) from prepro-ET-1. ET-1 is translated as an inactive precursor protein called prepro-ET-1. Prepro-ET-1 is cleaved by dibasic pair-specific endopeptidases and converted to big-ET-1. Specific processing of big-ET-1 by endothelin-converting enzymes (ECEs) results in the production of mature ET-1. (**B**) Ligand preference and signal transduction of ET_A_ and ET_B_ receptors. There are three distinct ET family peptides: ET-1, ET-2, and ET-3. The ET_A_ receptor has a ligand preference for ET-1 and ET-2, whereas the ET_B_ receptor binds these three ET ligands with a similar affinity. Both ET_A_ and ET_B_ receptors are linked to Gq- and G_12/13_-type G proteins, which activate Ca^2+^/protein kinase C (PKC) and rho/Rho-associated protein kinase (ROCK), respectively. ET_A_ receptors are also linked to the Gs protein to trigger cAMP-mediated signals, whereas ET_B_ receptors are linked to Gi to suppress them.

ET-1 biosynthesis is regulated by transcription and processing after translation of the prepro-ET-1 gene, which is a precursor protein. Transcription of ET-1 is promoted by transcription factors including AP-1, GATA-2, Smad, HIF-1α, and NFkB [50], for which the binding sites are present in the 5′-flanking region of the gene. These transcription factors are activated not only by cytokines and hormones but also by pathological conditions such as hypoxia [51] and mechanical stress [16], which underlie the increase in ET-1 production under pathological conditions. The prepro-ET-1 protein is cleaved by neutral endopeptidase to form an inactive precursor called big-ET (Figure 1A). The conversion of big-ET to ET-1 is mediated by a family of endothelin-converting enzymes (ECEs). ECEs have three isoforms, ECE-1, ECE-2, and ECE-3, which differ in cell distribution, localization, and substrate specificity [17]. ECE-1 and ECE-2 are the prominent isoforms that cleave big-ET-1. These ECEs are involved in the production of ET-1 and the degradation of amyloid-β proteins [52,53], which is a causative factor of AD. Therefore, the role of ECEs in AD pathology has been investigated from the perspective of ET production and degradation of amyloid-β proteins [14,54].

### 2.2. Endothelin Receptors

ETs exhibit their actions through two G-protein-conjugated receptor subtypes: the ET_A_ and ET_B_ receptors. Among the endogenous ET ligands, the ET_A_ receptor shows higher affinities for ET-1 and ET-2 than for ET-3. However, the ET_B_ receptor has an equal affinity for these three ET ligands (Figure 1B) [17]. Both of these ET receptors are linked to the Gq protein and increase intracellular Ca^2+^ by activating phospholipase C (PLC) [55,56,57]. For adenylate cyclase-mediated signals, the ET_A_ and ET_B_ receptors have different regulatory mechanisms, wherein the ET_A_ type is Gs-linked to increase cAMP, whereas ET_B_ is linked to Gi and suppresses the signal [58,59]. In addition, the ET_A_ and ET_B_ receptors are linked to the G_12/13_ protein. Signals triggered by the G_12/13_ protein activate the Rho protein, a low molecular weight G protein, and stimulate Rho-associated protein kinase (ROCK) [60], which regulates cellular proliferation, Ca^2+^, and cytoskeletal actin reorganization [61,62,63,64]. Similar to other G-protein-coupled receptors, ET receptors form dimers. Evance et al. showed that the ET_A_/ET_B_ heterodimer induces a long-lasting intracellular Ca^2+^ increase in response to ET-1, which was not observed by the activation of ET_A_ or ET_B_ receptor homodimers [65,66].

ET receptors are also expressed in astrocytes. Ligand binding experiments [17] and measurement of mRNA expression levels [16] showed that ET_B_ receptors are highly expressed in astrocytes over ET_A_ receptors. Activation of the astrocytic ET_B_ receptor causes increased intracellular Ca^2+^ and activation of protein kinase C/extracellular signal-regulated kinase signals via Gq-type proteins [17,64,67,68], which are intracellular signals involved in ET-induced astrocytic proliferation [64,69,70] and the production of some bioactive substances [71,72,73]. The astrocytic ET_B_ receptor was shown to be coupled with Gi-type proteins. The astrocytic ET_B_-receptor-mediated Gi signal was reported to be involved in ET-induced reduction in intercellular communication through gap junctions [74]. Rho protein-mediated signals can be activated by astrocytic ET_B_ receptors [63]. Activation of the ET_B_-receptor-mediated Rho signal in astrocytes involves cytoskeletal reorganization [63,75] and cell-adhesion-dependent proliferation [64,76]. As astrocytic proliferation and morphological alteration occur with phenotype conversion to reactive astrocytes [3,4], these signal mechanisms triggered by ET_B_ receptors are thought to underlie the induction of reactive astrocytes.

### 2.3. Endothelin Agonists and Antagonists

Due to the potent vasoconstricting action of ET-1, studies on ET receptor agonists and antagonists have been directed toward application in medications for cardiovascular diseases [17] (Table 1). BQ123 [77] and FR139317 [78] are peptide-based ET_A_-selective antagonists that are generated shortly after the discovery of ET-1. However, these ET_A_ antagonists are unsuitable for oral administration because of the low gastrointestinal absorption due to the peptide structure and have not been clinically applied. Conversely, bosentan (ET_A_/ET_B_ antagonist) [79], ambrisentan (ET_A_ selective antagonist) [80], and macisentan (ET_A_/ET_B_ antagonist) [81], which are orally administered, are approved in many countries for the treatment of pulmonary arterial hypertension. As an ET receptor agonist, IRL-1620, which shows high selectivity for the ET_B_ receptor, was developed [82]. IRL-1620 was reported to ameliorate ischemic brain injury and is expected to be a novel neuroprotective drug [83,84]. BQ788 [85], A192621 [86], and IRL-2500 [87] are selective ET_B_ receptor antagonists. Nagiri et al. suggested that IRL-2500 acts as an inverse agonist by analyzing the structure of the IRL-2500/ET_B_ receptor complex [88]. However, to date, only a few studies have attempted to examine the roles of ET_B_ receptors in the treatment of diseases.

## 3. ET System in the Brain

Increases in brain ET-1 have been observed in animal models of nerve injury [18,19,24] and in patients with stroke, head trauma, and neurodegenerative diseases [20,21,22,23,25]. Immunohistochemical observations of a damaged brain have shown that ET-1 is produced by brain microvascular endothelial cells and reactive astrocytes. Factors such as tumor necrosis factor-α, interleukin-1β and thrombin, as well as hypoxia induce ET-1 production in brain microvascular endothelial cells [89] and astrocytes [90,91,92]. ET-1 stimulates astrocytic ET-1 production. We found that the stimulation of ET_B_ receptors in mouse cultured astrocytes increased prepro-ET-1 mRNA levels [24]. Furthermore, prepro-ET-1 expression in the mouse TBI model was reduced by the antagonism of ET_B_ receptors [24]. These results indicate that astrocytic ET-1 production in brain disorders is enhanced by an autocrine mechanism using ET-1.

Both ET_A_ and ET_B_ receptors are present in the brain with different cellular distributions. Brain ET_A_ receptors are expressed in vascular smooth muscle. The activation of brain ET_A_ receptors causes vasospasm in several brain disorders, which aggravates ischemic brain damage [93,94]. In the brain, ET_B_ receptors are highly expressed, especially in astrocytes. Astrocytic ET_B_ receptors are upregulated and accompanied by the conversion to reactive astrocytes [41,95,96,97]. Together with an increase in ET-1, the upregulation of astrocytic ET_B_ receptors suggests that ET_B_-mediated regulation of astrocytic function becomes more pronounced in brain disorders.

## 4. ET_B_-receptors-mediated Astrocytic Activation

Phenotypic conversion of resting astrocytes to reactive astrocytes is commonly observed in various brain disorders. Since reactive astrocytes affect brain damage and/or the recovery of the damaged nervous system, mechanisms to induce reactive phenotype conversion have been investigated. Administration of ET_B_ agonists into the rat brain increased the number of GFAP-positive hypertrophic astrocytes without neuronal degeneration or microglial activation [39]. In animal models of stab wound injury [40] and brain ischemia [41], the induction of activated astrocytes was reduced by administration of the ET_B_ antagonist BQ788. With the conversion of the activated phenotype, astrocytes become hypertrophic and proliferative [3,4]. Stimulation of ET_B_ receptors in cultured astrocytes causes morphological alterations accompanied by cytoskeletal actin reorganization [63,98] and stimulated cellular proliferation [64,69,70], which is consistent with the action of ET_B_ receptors in vivo. These findings indicate that the activation of astrocytic ET_B_ receptors promotes their conversion to activated astrocytes. The conversion to reactive astrocytes is induced by several signaling factors in the damaged nerve tissues [3]. Among these factors, the ET_B_-receptor-mediated mechanism is characterized by maintaining or enhancing the activated state of astrocytes through the autocrine mechanism of ET-1, suggesting a pivotal role for ET_B_-mediated induction of reactive astrocytes.

**Figure 2:**
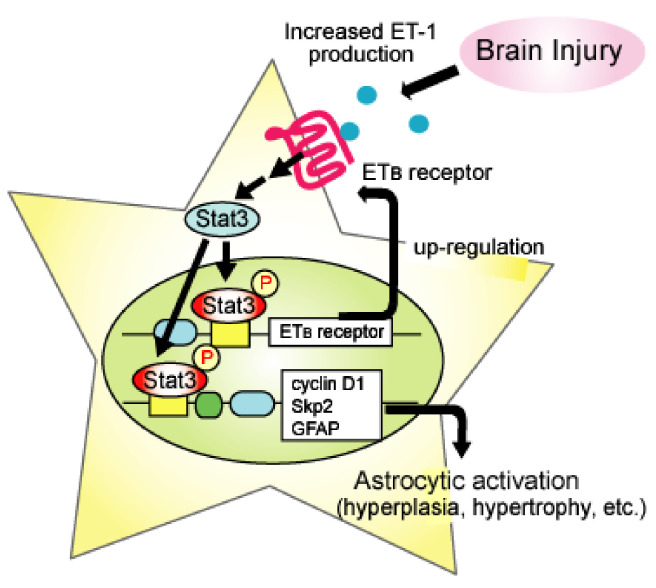
Positive feedback mechanism of the astrocytic ET_B_ receptor signal through activation of Stat3. Activation of astrocytic ET_B_ receptors stimulates the transcription of cyclin D1, S-phase kinase-associated protein 2 (Skp2), and GFAP through Stat3. Increases in cyclin D1, skp2m, and GFAP proteins are involved in proliferation and hypertrophy associated with phenotype conversion to reactive astrocytes. Activated Stat3 also promotes transcription of ET_B_ receptors. The upregulation of ET_B_ receptors results in enhancement in Stat3-mediated gene expression in astrocytes.

Stat3, a transcription factor, is activated in response to brain injury, where reactive astrocytes have high levels of activated Stat3 [99,100,101]. Increased production of GFAP, which underlies the hypertrophy of activated astrocytes, is stimulated by gene transcription through Stat3. In addition, cyclin D1 and S-phase-regulated kinase-2, which are proteins that promote cell cycle G1/S phase transition and are upregulated in activated astrocytes [102,103,104], have a Stat3 binding site on their gene promoter. Studies have shown that the inhibition of Stat3 prevents the induction and proliferation of reactive astrocytes in animal models of brain injury [105,106]. We showed that the activation of ET_B_ receptors in cultured astrocytes activated Stat3 and stimulated the transcription of cyclin D1 and Skp2 [69]. Administration of BQ788 reduced the activated form of Stat3 in a mouse model of TBI [69]. This result indicates that the generation of activated astrocytes by ET_B_ receptor stimulation is Stat3-mediated. LeComte et al. showed that the transcription of ET_B_ receptors was promoted by Stat3, suggesting that this mechanism underlies the upregulation of astrocytic ET_B_ receptors in brain ischemia [95]. Thus, it can be concluded that ET_B_-receptor-mediated Stat3 activation is an intracellular signal involved not only in astrocyte phenotype conversion but also in the positive feedback mechanism due to ET_B_ receptor upregulation (Figure 2).

## 5. Roles of Astrocytic ET_B_ Receptors in Brain Disorders

As reactive astrocytes are involved in the pathogenesis of many brain disorders, examinations have been conducted to improve brain disorders by controlling the astrocytic functions. Studies using animal models of brain disorders suggest that ET_B_-receptor-mediated alterations of astrocytic functions have a beneficial effect on AD, brain ischemia, neuropathic pain, and TBI.

### 5.1. Alzheimer’s Disease

Reactive astrocytes are observed in the brains of patients with AD and contribute to the pathology of AD [14]. In AD, the accumulation of amyloid-β proteins in the brain is the cause of nerve cell degeneration and cognitive impairment. In addition, ischemic neurodegeneration associated with decreased cerebral blood flow is involved in the progression of cognitive injury due to AD. In the brains of AD patients, ET-1 content and ECE-2 expression were increased [26]. Hung et al. showed that astrocyte-specific overexpression of ET-1 promoted amyloid-β production [107]. Conversely, in cultured neurons, amyloid-β proteins increase ECE expression and ET-1 production [108]. These observations suggest that ET-1 and amyloid-β proteins promote their expression and aggravate AD pathology. It has been suggested that ECE also cleaves amyloid-β proteins and removes them from the brain [109]. Therefore, further investigation into the pathological significance of increased ECE expression in AD is necessary. It was shown that the dysfunction of brain blood vessels and reduction in cerebral blood flow are observed in the brains of patients with AD. Since reduction in cerebral blood flow correlates with the degree of cognitive symptoms, it is considered a factor aggravating AD pathology. In addition to direct action on neuronal cells, the accumulation of amyloid-β proteins causes cerebrovascular injury and reduction in cerebral blood flow. Elesber et al. showed that the non-selective ET receptor antagonist bosentan improves cerebrovascular dysfunction in AD model mice overexpressing amyloid precursor protein [110]. In the pathological brain, ET-1 production occurs in activated astrocytes, which causes a decrease in cerebral blood flow. Therefore, suppression of astrocyte activation in the AD brain is expected to produce beneficial effects by improving cerebral blood flow and by reducing amyloid-β production.

### 5.2. Brain Ischemia

**Figure 3:**
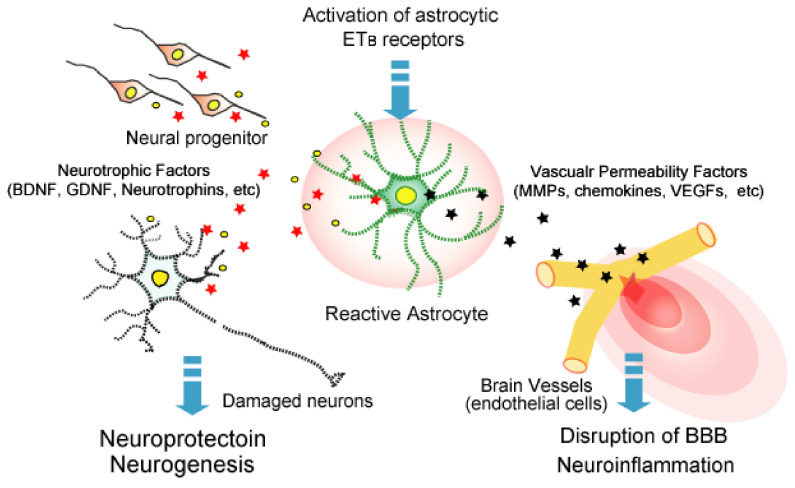
Roles of ET_B_ receptor-mediated bioactive factor production from reactive astrocytes. Increased ET-1 in brain disorders activates astrocytic ET_B_ receptors and induces reactive astrocytes. Reactive astrocytes release various types of bioactive factors. These include factors that increase the permeability of brain microvessels to promote blood–brain barrier (BBB) disruption and neuroinflammation, and factors that promote nerve protection and neurogenesis.

Due to its potent vasoconstricting action, the role of brain ET-1 was examined in relation to the vasospasm observed during cerebral ischemia. Since ET_A_ receptors mediate the vasoconstricting action of ET-1, the effects of ET_A_ antagonists on nerve damage in animal models of brain ischemia have been examined [93,94]. Although these animal experiments indicate the protective effect of ET_A_ antagonists, these drugs have not been clinically applied for cerebral ischemic injury at the time of writing. In contrast, Leonard et al. reported that the ET_B_ receptor agonist IRL-1620 had a neuroprotective effect and promoted the recovery of nerve function in a rat brain ischemia model using middle cerebral artery occlusion [84]. IRL-1620 administration was accompanied by angiogenesis and neurogenesis, which in turn were accompanied by increased vascular endothelial growth factor (VEGF) and nerve growth factor (NGF) production [83]. Activation of astrocytic ET_B_ receptors stimulates the production of several growth factors to induce angiogenesis and neurogenesis, including brain-derived neurotrophic factor (BDNF), glial cell line-derived neurotrophic factor (GDNF), VEGF, and NGF (Figure 3) [72,111,112,113]. Although it was not examined if the protective action of IRL-1620 against cerebral ischemia is caused by astrocyte-derived growth factors, these observations suggest that the activation of astrocytic ET_B_ receptors is beneficial in promoting the recovery of nerve functions after brain ischemia.

### 5.3. Neuropathic Pain

ET-1 is known to modulate nociceptive pain by affecting neurotransmission in the ascending pain pathway [114]. In the spinal cord, ET-1 induces hyperalgesia via the activation of ET_A_ receptors. ET-1 also has an anti-nociceptive effect, which was reported to be mediated through ET_B_ receptors [114]. The modulation of transmission in the pain pathway by ET-1 is mediated by the regulation of ion channel activity and neurotransmitter release in the spinal cord and brain. Neuropathic pain is an intractable pain that occurs without tissue damage.

The induction of neuropathic pain is caused by hyperexcitation of neural networks in the spinal pain pathway. Although the mechanisms underlying hyperexcitation in the pain pathway have not been fully clarified, previous studies showed the involvement of reactive astrocytes in the spinal cord. Increased numbers of reactive astrocytes were observed in the spinal cord of experimental animals with neuropathic pain [115]. Tsuda et al. showed that administration of a Stat3 inhibitor to a rat neuropathic pain model resulted in the suppression of the development of reactive astrocytes in the spinal cord and recovery from the established hyperalgesia [116]. Yamasaki et al. found that, in neuropathic pain induced by allergic inflammation, astrocytic ET_B_ receptors were upregulated with the induction of reactive astrocytes in the spinal cord [117]. The suppression of reactive astrocytes by BQ788 also reduced hyperalgesia [117]. These observations suggest that the ET_B_-receptor-mediated induction of reactive astrocytes causes neuropathic pain.

### 5.4. Traumatic Brain Injury (TBI)

TBI is often caused by a physical blow to the brain in a traffic accident or a fall. In the acute phase of TBI, disruption of the blood–brain barrier (BBB) occurs around the impact core region. Disruption of the BBB allows entry of blood components and blood cells into the brain parenchyma, which causes brain edema and neuroinflammation that aggravates brain damage due to TBI. Therefore, BBB protection in the acute phase is critical for the recovery of patients with TBI. A large part of the BBB is formed between the brain microvascular endothelial cells and astrocytes. The permeability of brain microvessels is not static and is not regulated by various factors produced by astrocytes (Figure 3) [118]. In the acute phase of TBI, reactive astrocytes release excessive amounts of vascular permeability regulators, such as VEGF and matrix metalloproteinases (MMPs), to brain microvessels and disrupt the barrier function [13]. In a mouse fluid percussion injury (FPI)-induced TBI model, we found that BQ788 ameliorated the disruption of BBB and brain edema accompanied by a decrease in reactive astrocytes [36]. Amelioration of BBB disruption was also achieved by the administration of bosentan (ET_A_/ET_B_ antagonist) after FPI to the mouse brain [24]. These findings indicate that ET_B_ antagonism is effective in reducing TBI-induced BBB disruption. Activation of astrocytic ET_B_ receptors stimulates VEGF and MMP9 production, while decreasing angiopoietin-1, a factor that stabilizes the BBB (Table 2) [119,120]. In the mouse TBI model, BQ788 normalized the altered expressions of VEGF, MMP9, and angiopoietin-1 in the injured region [42,121]. The actions of these vascular permeability regulators are thought to underlie protection of the BBB by ET_B_ receptor antagonism. BBB disruption by TBI involves several factors that are produced by reactive astrocytes. The antagonism of ET_B_ receptors simultaneously improves the production of these factors by reducing astrocytic activation, which suggests that ET_B_ antagonists effectively protect BBB function in the acute phase of TBI.

## 6. Conclusions

As the pathological roles of astrocytes have been clarified, therapeutic strategies targeting reactive astrocytes are being studied in several brain disorders. Astrocytic activation is triggered by the release of several signaling molecules from damaged nerve tissues (Table 2). Among these factors, the ET-1/ET_B_ receptor system maintains and enhances the activated state of astrocytes through the autocrine pathway of ET-1 and upregulation of ET_B_ receptor expression. This positive feedback mechanism indicates a pivotal role of the ET-1/ET_B_ receptor system in the functional regulation of reactive astrocytes. In addition, many ET receptor agonists and antagonists have been developed, some of which are clinically applied. In the future, ET receptor agonists and antagonists are expected to be clinically applied in the treatment of several brain disorders.

## Figures and Tables

**Table 1:** Agonists and antagonists for ET receptors.

	Agonist	Antagonist
ET receptor non-selective	ET-1	Bosentan, Macitentan
ET_A_ selective	sarafotoxin 6b	Ambrisentan, Sitaxsentan, Atrasentan, Clazosentan, Zibotentan, S-0139, SB234551, Ro-61-1790
ET_B_ selective	sarafotoxin 6c, IRL-1620, BQ3020, Ala^1,3,11,15^-ET-1	BQ788, IRL-2500, A192621, RES-701-1

**Table 2:** Regulations of astrocytic bioactive factors by ET_B_ receptors.

	Neurotrophic Factors	Vascular Permeability Regulators	Others
Up-regulation	GDNF [72], BDNF [111], NGF [112]	VEGF [119], MMP2 [120], MMP3 [73], MMP9 [71,120] ET-1 [24]	CCL2/MCP-1 [122], CXCL1/CINC-1 [122]
Down-regulation		angiopoietin-1 [119,121] sonic hedgehog [123]	CX3CL1/fractalkine [122], ephrin-A2, -A4, -B2, -B3 [124]

GDNF, glial cell line-derived neurotrophic factor; BDNF, brain-derived neurotrophic factor; NGF, nerve growth factor; VEGF, vascular endothelial growth factor; MMP, matrix metalloproteinases; ET-1, endothelin-1; MCP-1, monocyte chemotactic protein-1; CINC-1, cytokine-induced neutrophil chemoattractant-1.
